# The Association Between Chemerin Levels and Gestational Diabetes Mellitus: An Updated Systematic Review and Meta-Analysis

**DOI:** 10.3390/ijms26146622

**Published:** 2025-07-10

**Authors:** Hitham Aldharee, Yousif R. Makki, Hamdan Z. Hamdan

**Affiliations:** 1 Department of Pathology, College of Medicine, Qassim University, Buraidah 51452, Saudi Arabia; h.abualbasher@qu.edu.sa; 2 Department of Diabetes Center, Medical City, Qassim University, Buraidah 51452, Saudi Arabia; 3 Department of Community Medicine and Public Health, West Kordofan University, Al-Fulah 11111, Sudan; alblela133010@gmail.com

**Keywords:** chemerin, diabetes mellitus, gestational diabetes mellitus, pregnancy, hyperglycemia, normoglycemia

## Abstract

Chemerin is an adipokine that is associated with insulin resistance, a feature well marked in gestational diabetes mellitus (GDM). Recent publications and meta-analyses investigating chemerin levels in GDM remain inconclusive. This updated systematic review and meta-analysis aims to update the current evidence of an association between chemerin and GDM. The databases PubMed, ScienceDirect, and Google Scholar were searched for eligible articles from their inception up to 1 April 2025. Pooled standardized mean differences (SMDs) and 95% confidence intervals (CIs) of the chemerin levels between GDM cases and normoglycemic controls were calculated using the “meta” package in “R” software. Twenty-two studies were included in this meta-analysis, comprising a total of 1735 GDM cases and 1701 normoglycemic pregnant controls. Due to significant heterogeneity, a random effects model was applied, and the chemerin levels were found to be significantly higher in cases compared to normoglycemic controls [SMD = 0.97, 95% CI (0.16; 1.78) ng/mL; *p* = 0.020]. Subgroup analysis showed that studies conducted in Asia, studies utilizing a case–control design, patients younger than 30 years, and patients with a BMI less than 28 showed significantly higher chemerin levels in cases compared to controls. Meta-regression analysis indicated that only patients over 30 years old showed a negative association with chemerin levels. No evidence of publication bias was observed. This updated meta-analysis confirmed that chemerin levels are elevated in cases of GDM, which may indicate its involvement in the pathogenesis of GDM. Further longitudinal studies are needed to consolidate this finding.

## 1. Introduction

Gestational diabetes mellitus (GDM) is a common metabolic disorder that complicates around 7.1% to 27.6% of pregnancies worldwide [1]. It is characterized by glucose intolerance occurring during pregnancy in women who were previously normoglycemic [2]. GDM increases the risk of type 2 diabetes mellitus for both the mother and her offspring later in life [3].

To date, no definite cause of GDM has been identified; however, several risk factors have been reported to be associated with GDM development, including maternal obesity, oxidative stress, genetic factors, and insulin resistance [4,5,6,7].

Chemerin is an adipokine encoded by a gene known as Retinoic Acid Receptor Responder 2 (RARRES2; OMIM #605139). It is part of a group of cytokines primarily synthesized and secreted by adipose tissue [8]. In addition to their roles in regulating cellular energy levels, insulin secretion, and appetite control, chemerin is thought to be involved in promoting the inflammatory process and developing insulin resistance [4]. Moreover, numerous studies indicate that changes in RARRES2 gene expression and elevated chemerin levels are associated with diabetes and obesity, and potentially with GDM as well [9,10,11,12,13,14]. It is widely accepted that insulin resistance and β-cell dysfunction contribute to GDM pathogenesis [15]. Additionally, many studies have documented a positive correlation between chemerin levels and indices of insulin resistance in cases of GDM [16,17], suggesting the possible involvement of chemerin in GDM pathogenesis.

Chemerin levels do not significantly differ between individuals with type 2 diabetes and normoglycemic controls. However, significantly higher chemerin levels have been observed in normoglycemic obese individuals compared to normoglycemic non-obese subjects [18,19]. During normal, non-obese pregnancy, chemerin levels progressively increase with gestational age, reaching their highest levels in late pregnancy. Nevertheless, when compared to non-pregnant healthy controls, this increase does not reach statistical significance levels [20]. When comparing chemerin levels among obese pregnant women with GDM and those with GDM who are lean or overweight, no significant differences were observed [9]. However, reports regarding the association between chemerin levels and GDM are conflicting. Some studies have reported increased chemerin levels in GDM cases [12,21], while others have reported lower levels [13,22], and a few have found no significant difference [9,10,23]. Moreover, two previous meta-analyses investigated the association between chemerin levels and GDM and reported different findings. The first meta-analysis by Zhou et al. [24] reported significantly elevated levels of chemerin in GDM cases compared to controls. In contrast, the latest meta-analysis by Sun et al. [25] found no significant difference.

Given the potential pivotal role of chemerin in the pathophysiology of GDM—particularly its unique association with insulin resistance and its distinct expression pattern during pregnancy—and considering that several new studies have been published since the last review, this study aims to provide an updated synthesis of the current evidence regarding the association between chemerin levels and GDM. Such an update is essential for healthcare providers and researchers to enhance our understanding of GDM pathogenesis and to inform the development of effective management strategies.

## 2. Methods

### 2.1. Study Protocol and Search Strategy

In this systematic review and meta-analysis, we adhered to the Preferred Reporting Items for Systematic Reviews and Meta-Analyses guidelines [26]; see Table S1. We searched online databases, including PubMed, Google Scholar, and ScienceDirect, for published articles that measured chemerin levels during pregnancy in women with GDM and compared these levels to those of normoglycemic pregnant controls.

The search process utilized both MeSH terms and non-MeSH terms in combination with Boolean operators [OR] and [AND]; see Table S2. It was conducted from inception until the first of April 2025. The search strategy and keyword selection were developed based on the Population Intervention Comparison Outcome and Study design (PICOS) framework [27]:

P (population): pregnant women OR pregnancy.

I (intervention/exposure): chemerin OR Retinoic Acid Receptor Responder 2.

C (comparison): normoglycemic OR healthy pregnant OR normal pregnant.

O (outcome): gestational diabetes OR gestational diabetes mellitus OR GDM

S (study design): case–control OR cohort OR cross-sectional.

Two researcher reviewers (HA and YRM) independently evaluated and selected eligible studies for this meta-analysis following initial screening and reading. Any disagreements were resolved through discussions with the judging researcher (HZH); see (Table 1).

### 2.2. Inclusion Criteria

The articles were eligible for inclusion if they measured chemerin levels in serum or plasma samples during the pregnancy period in cases of GDM and compared them to normoglycemic controls; reported chemerin levels as mean (standard deviation) or expressed them in other forms that can be converted to mean (SD); and were designed as case–control, cohort, or cross-sectional studies. No language restrictions were applied to the articles.

### 2.3. Exclusion Criteria

Studies were excluded if they did not report chemerin concentrations in serum or plasma. Articles with the following formats were excluded: letters to editors, case reports, reviews, and animal studies. Studies that did not present chemerin levels in mean (SD) or any convertible format were also excluded.

### 2.4. Definition of the Outcome of Interest

The primary outcome of this meta-analysis is the association between chemerin levels and GDM. This association was evaluated by calculating the standardized mean difference between GDM cases and normoglycemic controls. GDM was defined according to the criteria of Coustan and Carpenter as the presence of two or more values exceeding the following thresholds: fasting plasma glucose > 95 mg/dL (or 1 h post-load glucose > 180 mg/dL, 2 h post-load glucose > 155 mg/dL, and 3 h post-load glucose > 140 mg/dL) [40].

### 2.5. Assessment of Risk of Bias

The risk of bias was assessed for each study using the Newcastle–Ottawa Scale (NOS) for case–control and cohort studies, while a modified version of the NOS was applied to cross-sectional studies [41]. The scale evaluates studies across three major areas: participant selection, comparability of study groups, and ascertainment of outcomes of interest. It employs a star-based scoring system with a maximum score of nine stars. Studies scoring ≥ 7 stars are considered high-quality, those scoring < 7 and ≥3 stars are deemed moderate-quality, and those scoring < 3 stars are classified as low-quality.

### 2.6. Data Extraction

Two reviewers (HA and YRM) retrieved the full articles of the selected studies, from which the following data were extracted: the last name of the first author, year of publication, country of the study, study design, commercial ELISA kit used, trimester at which chemerin levels were measured, the mean (SD) of chemerin in cases and controls, sample size, and the age and BMI of the cases and controls whenever applicable.

### 2.7. Statistical Analysis

The statistical analysis was performed using R software 4.4.0 (Puppy Cup) (The R Foundation for Statistical Computing, Vienna, Austria). The “meta (V7.0)” package [42] was used to calculate the pooled standardized mean difference (SMD) of chemerin between GDM cases and controls. Cochran’s *Q* test and Higgins index (*I*^2^) were used to evaluate heterogeneity in this meta-analysis. Cochran’s *Q* with *p* < 0.010 and *I*^2^ > 50% were considered indicative of heterogeneity in the included studies; accordingly, a random effects model shall be used; otherwise, a fixed effects model will follow [43]. Subgroup analyses were performed to identify sources of heterogeneity by grouping studies based on geographical continent, study design, and the trimester in which pregnant women were recruited and sampled. Meta-regression analysis was also computed to identify any possible factor(s) that impacted the pooled SMD of the chemerin.

Sensitivity analysis was performed to identify any study that significantly impacted the heterogeneity and overall effect. Publication bias was assessed visually using a funnel plot to identify the presence of any asymmetrical distribution among the studies, and quantitatively by performing Egger’s and Begg’s tests. A probability value < 0.05 was considered statistically significant.

## 3. Results

### 3.1. Study Selection

**Figure 1:**
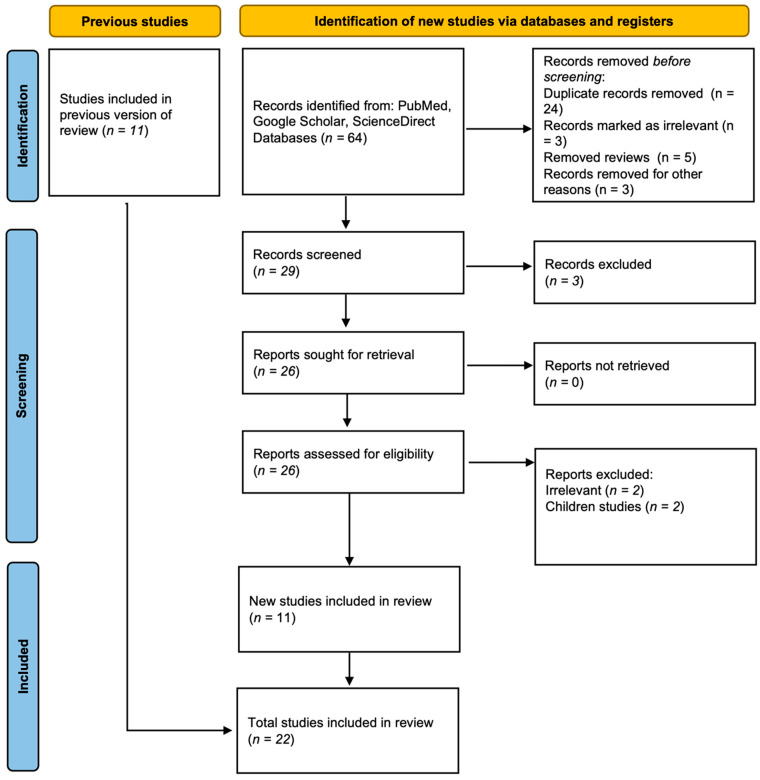
A flow chart demonstrating the study selection process.

The search across all databases initially retrieved 64 articles. After removing duplicates, review articles, case reports, and animal model studies, 29 articles remained eligible for screening. During the screening phase, three studies were excluded as they focused on other adipokines. Consequently, twenty-six articles were selected for full-text review, after which four additional studies were removed for reporting only expression results or being experimental studies. Ultimately, 22 studies were included in the systematic review and meta-analysis; see Figure 1.

### 3.2. Features of Selected Studies

A total of 22 studies were included in this systematic review and meta-analysis, comprising 1735 GDM cases and 1701 normoglycemic pregnant controls. According to the geographical location of these studies, thirteen were conducted in Asian countries: eight studies in China [13,14,16,17,30,33,38,40], two studies in Pakistan [12,21,22], one study in Malaysia, one study in Saudi Arabia [30], and one study in South Korea [35]. Six studies were conducted in European countries: three studies in Turkey [10,11,35], one in Greece [33], one in Germany [28], and one in Poland [38]. Two studies were conducted on the Australian continent [9,31], and only one study was conducted in North America [35] (Canada).

Ten studies measured chemerin levels during the second trimester only [10,11,12,21,34,35,36,37,38,39]; seven studies measured chemerin during the third trimester only [9,14,16,17,28,29,33]; two studies measured chemerin during both the second and third trimesters [22,30]; one study measured chemerin during the first and second trimesters [31]; and one study measured chemerin during the first and third trimesters [13]. Seventeen studies employed a case–control design [9,11,12,13,14,16,17,21,22,28,29,32,33,35,37,38,39], three utilized a cohort design [31,34,36], and two were cross-sectional studies [10,30], See Table 1.

### 3.3. Meta-Analysis Results

**Figure 2:**
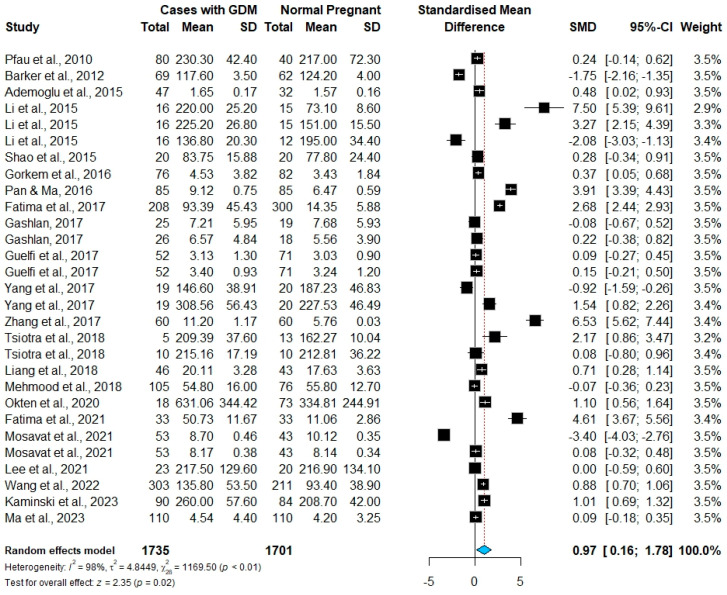
A forest plot of the included studies in the chemerin GDM meta-analysis [9,10,11,12,13,14,16,17,21,22,28,29,30,31,32,33,34,35,36,37,38,39].

This meta-analysis showed that the pooled SMDs of chemerin levels were significantly higher in pregnant women with GDM than in normoglycemic pregnant controls [SMD= 0.97, 95% CI (0.16–1.78) ng/mL; *p* = 0.020]; see Figure 2. The Higgins index (*I*^2^ = 98%) and Cochran’s *Q* test (*p* < 0.0001) both indicated significant heterogeneity among the included studies. Therefore, a random effects model was employed; see Figure 2.

**Figure 3:**
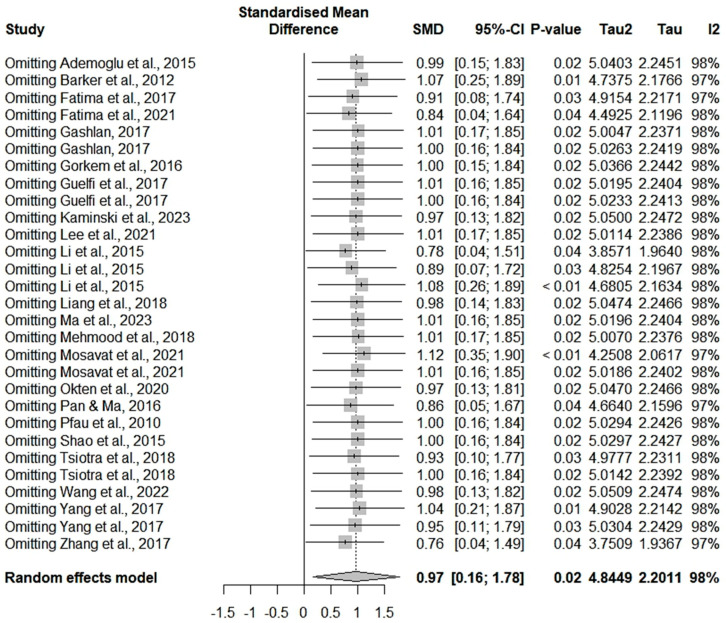
A sensitivity analysis of the included studies in the chemerin GDM meta-analysis [9,10,11,12,13,14,16,17,21,22,28,29,30,31,33,34,35,36,37,38,39].

To investigate potential sources of significant heterogeneity, we conducted a sensitivity analysis by re-running the meta-analysis while excluding one study at a time. The exclusion of individual studies did not substantially alter the overall estimate of the meta-analysis, nor did it reduce the measures of heterogeneity; see Figure 3.

**Figure 4:**
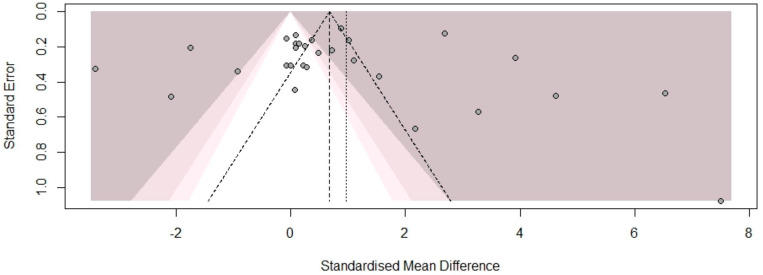
A funnel plot to detect the publication bias in the studies included in the meta-analysis.

No asymmetry was observed in the funnel plot, indicating the absence of publication bias (see Figure 4). This finding was further confirmed quantitatively by Egger’s test (t = 0.24, df = 27, *p* = 0.809) and Begg’s test (z = 1.26, *p* = 0.208), which showed no evidence of publication bias.

### 3.4. Subgroup Analysis and Meta-Regression

The included studies were stratified into subgroups to identify potential sources of heterogeneity. The selected studies were categorized into two groups based on the average age of the patients: ≥30 years and <30 years. The group aged <30 years revealed higher chemerin levels in cases with GDM than in controls [SMD = 2.31 (95% CI (0.82; 3.79); *p* < 0.01], while no significant difference was observed in the group aged ≥30 years (see Table 2). Additionally, we grouped the studies according to the average patients’ BMI into ≥28 kg/m^2^ and <28 kg/m^2^ groups. Only the group with BMI < 28 kg/m^2^ showed higher chemerin levels in GDM cases compared to the control group [SMD = 1.30 (95% CI (0.24; 2.35); *p* = 0.021] (see Table 2). We also conducted a subgroup analysis based on the types of commercial ELISA kits used to measure chemerin levels. Specifically, eight studies used R&D Systems kits, five used Biovendor, three used HCB, three used Millipore, two used Glory Bioscience, two used Elabscience, one used TaKaRa Biotechnology, one used Shanghai Korain Biotech, and one used Meilian. Additionally, three studies did not report the name of the ELISA kit used. Despite this stratification, substantial heterogeneity persisted across all subgroups (see Table S3).

Additionally, the studies were divided based on the pregnancy period at which chemerin was measured. In this subgroup, the studies were split into two groups: first/second trimester and third trimester groups. No statistical significance was observed between these two gestational periods; see Table 2. Furthermore, the studies were sub-categorized according to study design into a case–control group and other design groups (including cross-sectional and cohort). The case–control study group demonstrated significantly higher chemerin levels in GDM cases compared to controls, while the other design groups showed no significant difference; see Table 2. Likewise, the studies conducted in Asia showed significantly higher chemerin levels in GDM cases than in controls, while the studies conducted in other continents showed no statistically significant difference; see Table 2.

Met-regression analysis showed that only patient aged ≥30 years significantly influenced the overall effect [estimation coefficient = −2.13 (95% CI (−3.82; −0.411); *p* = 0.015], while none of the investigated continuous variables (study’s year of publication, NOS score, and study sample size) nor the other categorical variables (study design, trimester of measuring chemerin, study continent, and patients’ BMI) had a significant impact on chemerin levels; see Table 3.

## 4. Discussion

The main finding of this updated meta-analysis is that the levels of chemerin were significantly higher in pregnant women with GDM than in normoglycemic controls. This finding aligns with the previous meta-analysis by Zhou and coworkers [24], yet it contradicts the latest meta-analysis by Sun and colleagues [25]. The current updated meta-analysis includes 22 studies, which is exactly double the 11 studies included in Zhou et al.’s study [24] and the 10 studies included in Sun et al.’s [25] meta-analysis. In this study, no evidence of publication bias was observed, and the sensitivity analysis revealed that no single study influenced the overall effect or impacted the heterogeneity levels, indicating that the obtained result is robust.

Subgroup analysis showed that the group of patients aged less than 30 years had significantly higher chemerin levels than the controls. Additionally, the meta-regression analysis results indicated that only the group of patients aged ≥30 had decreasing levels of chemerin twice that found in patients under 30 years, suggesting a negative correlation between chemerin and patients’ age. This finding is in line with two previous meta-analyses [24,25]. However, little is known about the relationship between patients’ age and chemerin levels. Therefore, more studies are needed to explore this relationship in depth.

In this study, subgroups with lower BMI showed significantly higher chemerin levels in cases with GDM compared to controls. This finding is in line with the previous meta-analysis by Zhou et al. [24]; however, it contradicts the latest meta-analysis by Sun and colleagues [25]. This is in spite of the fact that chemerin is synthesized and secreted primarily from the adipose tissue, and studies have demonstrated that maternal BMI is positively correlated with chemerin levels [9,11]. One of the possible explanations of our finding is that we use the gestational BMI as a measure of obesity, which is less sensitive and unlike other indices such as the waist-to-height ratio and waist-to-hip ratio, which can predict metabolic disturbances [44,45].

In this study, the trimester at which chemerin was measured did not differ significantly between the group of studies that measured chemerin in the third trimester and those that measured it in either the first or second trimester. A previous study reported that, in the second trimester, chemerin levels are higher than those in the third trimester [46]. Yet, others have reported that chemerin is higher in the third trimester [13]. It has also been reported that, in addition to adipocytes, chemerin is released by serum albumin, which decreases as pregnancy advances due to the shift in nutrients toward the growing fetus rather than the mother [24]. However, in this study, this association is not clear, and more research is needed to draw a conclusion on this.

As found in the previous meta-analysis [24], GDM cases in Asian studies showed significantly higher chemerin levels than controls. This finding may be due to variations in genetic makeup across different ethnic groups. A recent Chinese cohort study and an Iranian study showed that chemerin gene SNPs, such as rs4721 and rs17173608 polymorphisms, can increase the risk of GDM [37,47]. Moreover, it has been confirmed that these chemerin SNPs can affect levels of expression and circulating chemerin [37,48]. From another perspective, there are variations in dietary habits across different populations [49]. Experimental evidence showed that feeding mice a high-fat diet significantly affects the levels of chemerin [50]. Perhaps differences in maternal dietary habits may partially explain the variations in chemerin levels.

It is worth mentioning that all included studies used ELISA to measure chemerin levels. However, we observed wide variations in the reported chemerin concentrations across studies. This variability may be attributed to differences in the ELISA kits used. Specifically, nine different ELISA kits—each with distinct calibration standards and detection limits—were employed across the studies. Furthermore, some of these commercial ELISA kits are based on polyclonal antibodies [51]. Previous evidence has shown that ELISA kits used to measure irisin—an adipomyokine—based on polyclonal antibodies exhibited high cross-reactivity with other proteins, potentially inflating the measured irisin concentrations [52]. Although no such cross-reactivity has been reported for chemerin to date, this possibility remains to be investigated and could represent a potential source of variability and heterogeneity in chemerin measurements.

Adipokines have been proposed as predisposing factors in the pathogenesis of GDM [53] and other pregnancy complications such as pre-eclampsia [54]. Several reports have shown that chemerin, in particular, is strongly associated with insulin resistance [14,28,33], a hallmark feature of GDM. Evidence indicates that chemerin receptors are extensively expressed in various endocrine organs, including the pancreas, ovaries, and placenta, pointing to their biological activity in these organs [55]. Experimental studies have demonstrated that chemerin is involved in regulating β-cell function, which includes the release of insulin and glucose homeostasis [56]. Moreover, it has been reported that chemerin may interfere with glucose uptake, thereby potentially aggravating hyperglycemia in GDM [57]. From another perspective, chemerin, as a pro-inflammatory cytokine, acts as a chemoattractant chemokine, potentially inducing or promoting inflammation [58]. Additionally, chemerin levels are well correlated with other inflammatory cytokines such as IL-6 and TNF-α, which are known to be involved in GDM pathogenesis [59,60]. Collectively, these premises provide a rationale for proposing chemerin as a predisposing factor for GDM.

While most of these events are observed on the maternal side, altered chemerin levels also impact fetal development. It has been reported that elevated chemerin levels are associated with increased fetal growth, leading to higher birth weight, large-for-gestational-age infants, and the development of macrosomia, a common complication of GDM [61,62]. This observation is supported by a very recent experimental animal study by Zhou et al., 2024, which reported that elevated chemerin levels may promote fetal overgrowth and help neutralize placental oxidative stress [63]. On the other hand, another animal model study on GDM showed that chemerin aggregation may contribute to fetal cognitive disorder through the activation of the pyroptosis pathway in macrophages [64]. These contrasting findings imply that chemerin’s effect on fetal development may be dose-dependent.

Although this meta-analysis included 22 studies, providing a robust association between chemerin levels and GDM, and no publication bias was observed either through funnel plot visualization or quantitative tests, it has several limitations that need to be addressed for better interpretation. Firstly, the heterogeneity among the studies is significant, and the subgroup analysis did not resolve the sources of this heterogeneity. Secondly, the results of the subgroup analysis may be influenced by the number of studies allocated to each group rather than reflecting a true biological association. Thirdly, most of the included studies followed a case–control design, and therefore, causality cannot be inferred or deduced. Lastly, insulin resistance indices were not investigated in this study, as not all studies provided this data; hence, this important covariate cannot be computed.

## 5. Conclusions

In conclusion, this updated meta-analysis demonstrated that chemerin levels were significantly higher in cases of GDM compared to normoglycemic controls. Chemerin levels were found to be significantly higher in studies conducted in Asia, in studies with a case–control design, in patients younger than 30 years, and in patients with a BMI < 28 kg/m^2^. However, due to significant heterogeneity, a random-effects model was applied; therefore, the findings should be interpreted with caution. Further studies with longitudinal designs are needed to explore in depth the association between chemerin levels and GDM.

## Figures and Tables

**Table 1:** Characteristics of the included studies in the updated meta-analysis of chemerin and gestational diabetes mellitus.

Study	Country	Continent	Study Design	Sampling Trimester	No. Cases/No. Controls	Chemerin Mean (SD) in GDM Cases, ng/mL	Chemerin Mean (SD) in Controls, ng/mL	GDM Cases Mean Age, Years	GDM Cases Mean BMI, kg/m^2^	Commercial ELISA Kit	NOS Score
Pfau et al., 2010 [28]	Germany	Europe	Case–control	Third	80/40	230.3 (42.4)	217 (72.3)	33	24.9	BioVendor (Karasek, Czech Republic)	8
Barker et al., 2012 [9]	Australia	Australia	Case–control	Third	69/62	117.6 (3.5)	124.2 (4)	35.2	31.2	R & D systems (Minneapolis, MN, USA)	7
Ademoglu et al., 2015 [11]	Turkey	Europe	Case–control	Second	47/32	1.65 (0.17)	1.57 (0.16)	30.2	31.3	BioVendor (Karasek, Czech Republic)	6
Li et al., 2015 [14]	China	Asia	Case–control	Third	16/15	220 (25.2)	73.1 (8.6)	29.3	23	HCB(Vancouver, BC, Canada)	7
Li et al., 2015 [14]	China	Asia	Case–control	Third	16/15	225.2 (26.8)	151 (15.5)	29.3	27.1		7
Li et al., 2015 [14]	China	Asia	Case–control	Third	16/12	136.8 (20.3)	195 (34.4)	29.14	33		7
Shao et al., 2015 [29]	China	Asia	Case–control	Third	20/20	83.75 (15.88)	77.8 (24.4)	30	28.73	NA	6
Görkem et al., 2016 [10]	Turkey	Europe	Cross-sectional	Second	76/82	4.53 (3.82)	3.43 (1.84)	27.59	36.25	BioVendor (Karasek, Czech Republic)	6
Pan & Ma, 2016 [17]	China	Asia	Case–control	Third	85/85	9.12 (0.75)	6.47 (0.59)	28.1	23.6	NA	7
Fatima et al., 2017 [12]	Pakistan	Asia	Case–control	Second	208/300	93.39 (45.43)	14.35 (5.88)	27.3	24.83	Glory Bioscience (Shanghai, China)	8
Gashlan 2017 [30]	KSA	Asia	Cross-sectional	Second	25/19	7.21 (5.95)	7.68 (5.93)	32.4	33.4	Elabscience Company	7
Gashlan 2017 [30]	KSA	Asia	Cross-sectional	Third	26/18	6.57 (4.84)	5.56 (3.9)	33.4	34.1		6
Guelfi et al., 2017 [31]	Australia	Australia	RCT	First	52/71	3.13 (1.3)	3.03 (0.9)	34.4	26.9	R & D systems (Minneapolis, MN, USA)	6
Guelfi et al., 2017 [31]	Australia	Australia	RCT	Second	52/71	3.4 (0.93)	3.24 (1.2)	34.4	26.9		7
Yang et al., 2017 [13]	China	Asia	Case–control	First	19/20	146.6 (38.91)	187.23 (46.83)	26.84	22.74	R & D systems (Minneapolis, MN, USA)	7
Yang et al., 2017 [13]	China	Asia	Case–control	Third	19/20	308.56 (56.43)	227.53 (46.49)	26.84	25.67		7
Zhang et al., 2017 [32]	China	Asia	Case–control	First	60/60	11.2 (1.17)	5.76 (0.03)	29.13	38.68	TaKaRa Biotechnology (Kusatsu, Japan)	7
Tsiotra et al., 2018 [33]	Greece	Europe	Case–control	Third	5/13	209.39 (37.6)	162.27 (10.04)	29.8	26.9	Millipore (Burlington, MA, USA)	7
Tsiotra et al., 2018 [33]	Greece	Europe	Case–control	Third	10/10	215.16 (17.19)	212.81 (36.22)	27.7	36		7
Liang et al., 2018 [16]	China	Asia	Case–control	Third	46/43	20.11 (3.28)	17.63 (3.63)	31.89	21.52	NA	8
Mehmood et al., 2018 [34]	Canada	America	Cohort	Second	105/76	54.8 (16)	55.8 (12.7)	35	25.78	Millipore (Burlington, MA, USA)	8
Okten et al., 2020 [35]	Turkey	Europe	Case–control	Second	18/73	631.06 (344.42)	334.81 (244.91)	32.05	27.06	Shanghai Korain Biotech	8
Fatima et al., 2022 [21]	Pakistan	Asia	Case–control	Second	33/33	50.73 (11.67)	11.06 (2.86)	24.23	25.22	Glory Bioscience (Shanghai, China)	7
Mosavat et al., 2021 [22]	Malaysia	Asia	Case–control	Second	53/43	8.7 (0.46)	10.12 (0.35)	33.2	27	R & D systems (Minneapolis, MN, USA)	6
Mosavat et al., 2021 [22]	Malaysia	Asia	Case–control	Third	53/43	8.17 (0.38)	8.14 (0.34)	33.2	27		6
Lee et al., 2021 [36]	South Korea	Asia	Cohort	Second	23/20	217.5 (129.6)	216.9 (134.1)	31.8	23.1	BioVendor (Karasek, Czech Republic)	8
Wang et al., 2022 [37]	China	Asia	Case–control	Second	303/211	135.8 (53.5)	93.4 (38.9)	32.7	31.9	R & D systems (Minneapolis, MN, USA)	7
Kamiński et al., 2023 [38]	Poland	Europe	Case–control	Second	90/84	260 (57.6)	208.7 (42)	26.6	26.7	BioVendor (Karasek, Czech Republic)	7
Ma et al., 2023 [39]	China	Asia	Case–control	Second	110/110	4.54 (4.4)	4.2 (3.25)	32.24	28.99	Meilian ( Shanghai, China)	6

In 95% confidence interval, BMI: body mass index; NA: Not available; NOS: Newcastle–Ottawa Scale.

**Table 2:** Subgroup analysis of the chemerin levels in gestational diabetes mellitus.

Subgroup	Number of Studies	Number of GDM	Number of Controls	SMD (95% CI)	*I*^2^-Index
**Trimester**					
First/second	16	1274	1305	0.83 (−0.25; 1.91)	98.0%
Third	13	461	396	1.16 (−13; 2.45)	98.0%
**Continent**					
Asia	18	1131	1087	1.39 (0.12; 2.66) *	98.0%
Others	11	604	614	0.29 (−0.23; 81)	93.0%
**Study Design**					
Case–control	22	1376	1344	1.27 (0.20; 2.33) *	98.0%
Others	7	359	357	0.11 (−0.04; 0.27)	0.0%
**Patients’ age**					
≥30 years	16	1082	952	−0.05 (−0.57; 0.47)	95.0%
<30 years	13	653	749	2.31 (0.82; 3.79) *	98.0%
**Patients’ BMI**					
≥28 kg/m^2^	11	762	636	0.45 (−0.83; 1.73)	97.0%
<28 kg/m^2^	18	973	1065	1.30 (0.24; 2.35) *	98.0%

* statistical significance; BMI: body mass index; SMD: standardized mean difference; 95% CI: 95% confidence interval.

**Table 3:** Meta-regression analysis of the chemerin levels in gestational diabetes mellitus.

Covariate	Estimation Coefficient	Standard Error	*p*-Value	95% CI
NOS score	0.64	0.679	0.340	(−0.68; 1.97)
Year of publication	−0.02	0.196	0.928	(−0.40; 0.36)
Sample size	0.01	0.004	0.689	(−0.01; 0.02)
Trimester				
First/second	Reference	Reference		
Third	0.56	1.253	0.654	(−1.89; 3.01)
Continent				
Asia	Reference	Reference		
Others	−1.17	1.004	0.241	(−3.14; 0.79)
Study Design				
Case–control	Reference	Reference		
Others	0.75	1.085	0.487	(−1.37; 2.87)
BMI				
≥28 kg/m^2^	−0.48	0.972		
<28 kg/m^2^	Reference	Reference	0.618	(−2.38; 1.42)
Age				
≥30 years	−2.13	0.877		
<30 years	Reference	Reference	0.015	(−3.82; −0.411)

BMI: body mass index; 95% CI: 95% confidence interval; NOS: Newcastle–Ottawa Scale.

## Data Availability

The data used to generate the results in this manuscript are available in this manuscript.

## Supplementary Materials

The following supporting information can be downloaded at https://www.mdpi.com/article/10.3390/ijms26146622/s1.
